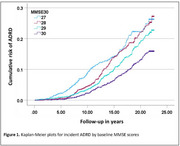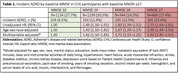# Risk of ADRD in older adults with MMSE ≥27 during 23‐year of follow‐up in the Cardiovascular Health Study

**DOI:** 10.1002/alz70857_102075

**Published:** 2025-12-24

**Authors:** Mo‐Kyung Sin, Richard M Allman, Charles Faselis, Annette L. Fitzpatrick, Fatemeh Choupani, Yuting Lin, Ali Ahmed

**Affiliations:** ^1^ Seattle University, Seattle, WA, USA; ^2^ Wake Forest University, Winston‐Salem, NC, USA; ^3^ DC VA, Washington, DC, USA; ^4^ University of Washington, Seattle, WA, USA

## Abstract

**Background:**

Impaired cognitive function, as measured by Mini‐Mental Status Examination (MMSE), is a risk factor for incident Alzheimer's disease and related dementias (ADRD). However, the association between MMSE within normal range (27‐30) and ADRD risk in older adults remains less understood.

**Method:**

Of the 5795 community‐dwelling adults aged ≥65 years in the de‐identified copy of the Cardiovascular Health Study (CHS), 4433 had a baseline normal cognitive function (MMSE 27–30). We compared baseline characteristics and incident ADRD in individuals with MMSE scores of 30, 29, 28 and 27. We then used a multivariable Cox proportional hazards model to examine the associations of MMSE scores of 29, 28 and 27 with incident ADRD, using MMSE 30 as the reference, and adjusting for 27 baseline covariates. ADRD was defined using ICD codes for principal or secondary hospital discharge diagnosis. Time to first ADRD diagnosis was used as time‐to‐event while patients without ADRD were censored at death or the end of study follow‐up, whichever occurred first.

**Result:**

Participants (59% women, 10% African American) with MMSE‐30 (*n* = 1228), MMSE‐29 (*n* = 1353), MMSE‐28 (*n* = 1079) and MMSE‐27 (*n* = 773) had mean ages of 71.1, 71.9, 72.6, and 73.4 years, respectively, and mean activities of daily living (ADL) impairment scores of 0.6, 0.8, 0.12 and 0.16, respectively (both *p* <0.001). Respective unadjusted incidence rates of ADRD were 8.5%, 11.4%, 12.5% and 13.6% during 23 years of follow‐up (average 14 years; Figure 1). When adjusted for age and the 26 other baseline characteristics (Table 1), including ADL, compared with MMSE 30, hazard ratios (95% confidence intervals) for incident ADRD in those with MMSE 29, 28, and 27 were 1.42 (95% CI,1.10–1.83; *p* = 0.006), 1.71 (95% CI, 1.32–2.23; *p* <0.001) and 1.92 (95% CI, 1.45–2.55; *p* <0.001), respectively. Other significant predictors included age, BMI, diabetes, depression, and smoking.

**Conclusion:**

In community‐dwelling older Americans with normal cognitive status, even a single point decline in MMSE score from 30 was associated with a significant, independent, and incremental risk of developing ADRD. MMSE could be used to risk‐stratify older adults with normal cognition for targeted ADRD prevention strategies.